# Molecular Biology in Glioblastoma Multiforme Treatment

**DOI:** 10.3390/cells11111850

**Published:** 2022-06-05

**Authors:** Claudia Abbruzzese, Michele Persico, Silvia Matteoni, Marco G. Paggi

**Affiliations:** 1 Cellular Networks and Molecular Therapeutic Targets, Proteomics Unit, IRCCS-Regina Elena National Cancer Institute, 00144 Rome, Italy; claudia.abbruzzese@ifo.it (C.A.); silvia.matteoni@ifo.it (S.M.); 2 Beth Israel Deaconess Medical Center, Harvard Medical School, Boston, MA 02215, USA; mpersic1@bidmc.harvard.edu

Glioblastoma (GBM, grade IV astrocytoma), the most frequently occurring primary brain tumor, presents unique challenges to therapy due to its location, aggressive biological behavior, and diffuse infiltrative growth, thus contributing to having disproportionately high morbidity and mortality. Radiotherapy plus concomitant and adjuvant temozolomide (TMZ) chemotherapy (the “Stupp protocol”) is the present standard of care for newly diagnosed GBM adult patients, aged under 70 years and in good general and neurological conditions [1]. Patients displaying O-6-methylguanine-DNA methyltransferase (MGMT) gene promoter methylation have a greater therapeutic benefit [2].

In GBM patients, relapse is almost always guaranteed, thus dictating patient prognosis. GBM growth and aggressiveness is not dependent on a single targetable factor because these tumors are highly heterogeneous and extremely adaptive [3,4,5]. Indeed, recurrent GBM is considered to arise from cell clones usually resistant to the previous therapeutic regimen. Currently, several drugs are utilized for recurrent GBM after first-line treatment, e.g., bevacizumab, regorafenib, and nitrosoureas, but no standard treatments have been clearly identified for the progressive disease yet.

With these premises, we can undoubtedly number GBM as one of the highest unmet medical needs. In fact, there is a strong need to increase our knowledge in several molecular mechanisms characterizing this tumor, as well as enriching our therapeutic arsenal. As a matter of fact, all the contributions made to this Special Issue go in this direction at the same time as making excellent contributions as research articles and review articles to Scientific literature as well.

Mauldin and collaborators [6] investigate the amount and functional status of tumor-infiltrating lymphocytes in a wide cohort of IDH1/2 wt GBM patients who were administered standard-of-care treatment for this disease. In multivariate analyses adjusted for clinical variables including age, resection extent, Karnofsky Performance Status (KPS), and MGMT gene promoter methylation status, the authors evaluated the overall survival (OS) correlation which was: (a) higher for patients displaying an elevated CD8+/CD4+ cellular ratio in the proliferative compartment (Ki67+); (b) lower for those displaying higher IFNγ amounts. To sum up, increased CD8+ tumor-reactive proliferating T cells and their ability to interfere with the immunoregulatory effects of IFNγ in the tumor microenvironment provide the patient with a better prognosis, as evaluated via OS.

Vàzquez Cervantes and collaborators [7] describe the importance of the kynurenine pathway in gliomatous tissue and the pivotal role played by kynurenine monooxygenase (KMO) in this context. The authors evaluated the KMO expression and activity in astrocytomas, which represents a peculiar trait that distinguishes brain tumor clinical samples from biopsies derived from patients with other neurological diseases. Their findings reveal that glial tumors, via their higher KMO activity, exploit metabolic and immune advantages, and that this factor—as well as its druggability—can represent a critical vulnerability in these malignancies, thus opening new therapeutic options.

Kraboth and colleagues [8] explore the role of DNA CpG methylation in modulating the expression of neurotransmitters in GBM. These molecules have a well-known role in CNS development and physiology but have been found to be equally important in GBM, due to their interplay with neurons and glioma cells [9,10]. Here, the authors analyze a GBM cohort consisting of 21 pairs of primary (GBM1) and recurrent GBM (GBM2) for selected catecholamine pathway markers, finding significant correlations in DRD2 and ADRBK1/GRK2 expression when comparing GBM1 and GBM2, while for ADRA1D, SLC18A2 a trend was predicted, but without reaching the statistical significance. At any rate, these findings support the active role these neurotransmitters and their receptors play in gliomagenesis. Being in the field of neuropharmacology, several drugs are able to interfere at different levels between neurotransmitters and their receptors; these findings pave the way for an active repositioning of neuropharmaceuticals in GBM therapy.

The paper by Fischer and collaborators [11] describes a computational approach for establishing drug effectiveness in GBM by comparing three well-known calculation methods on a collection of nine in vitro model systems exposed to a library of 231 clinical drugs. Despite the limitations that usually characterize in vitro studies, the impossibility of considering the effect of any single medication when added to the standard clinical care, i.e., surgery and radiotherapy plus temozolomide, helps this limit the number of possible candidate drugs for further preclinical and clinical studies.

Persico and colleagues [12] address the issue of repurposing antipsychotic drugs in GBM therapy, in view of the physicochemical and pharmacological characteristics of these drugs and on evaluating their effect on each of the ten hallmarks of cancer, as described by Weinberg and Hanahan in their seminal papers [13,14]. In addition, the authors outline the possibility for repurposed/repositioned drugs to swiftly enter clinical trials, thus cutting down both time and costs for these drugs to reach the patient’s bedside.

Batara and colleagues [15] delve into the role of autophagy in GBM cell death or survival. Autophagy, a feature that allows the recycling of older cellular structures, essentially used to induce the renewal of cellular organelles and bioenergetics purposes, characterizes normal and cancer cells. When in excess, autophagy, instead of being a cytoprotective tool, can become cytotoxic, bringing cells to death. Thus, autophagy-modulating compounds can generate an imbalance in cancer cells, which are often characterized by high autophagic levels at baseline and, therefore, unable to increase the autophagy rate further. Here, the authors accurately describe the molecular mechanisms involved in autophagy as well as the effect of several drugs known to modulate autophagy, pertinent to the effect of these modulators on glioma stem cells.

Lange and collaborators [16] discuss the role of glutamatergic signaling and its relationship with progression and malignancy in GBM. Epileptic seizures are often the onset symptom in GBM and are essentially due to several pathophysiological mechanisms where glutamate and other neurotransmitters play a key role. In this context, due to the frequent association between GBM and epilepsy, the authors describe the mechanisms of the action of various antiepileptic drugs, which, by interfering with neurotransmitters, might be beneficial for both the seizure syndrome and the clinical course of GBM.

Zhang & Lin [17] describe the role of the enzyme asparagine endopeptidase (AEP, legumain) in the progression of several tumors, e.g., breast carcinoma, GBM, gastric carcinoma, and ovarian carcinoma. The authors delve into the 3D structure of the enzyme and illustrate the role of AEP, which has become clearer following the identification of key cellular factors, i.e., p53, integrin αvβ3 and the proteases MMP-2 and MMP-9, as substrates of its endopeptidase activity. The last section of their work is dedicated to the possible role of AEP inhibitors acting as anticancer agents in combination with the first-line therapeutic approach specific for each of the cancer types discussed.

Shi and collaborators [18] describe the importance of the tumor microenvironment in cancer progression and focus on the role of Fibroblast Activation Protein (FAP) in evading immune surveillance in GBM patients. FAP expression appears upregulated in cancer and capable of promoting invasiveness via the suppression of the immune response toward GBM and the induction of resistance to TMZ. Thus, while FAP overexpression could represent an interesting diagnostic/prognostic marker in GBM, this factor also appears as an attractive therapeutic target to be considered in GBM therapy.

Overall, this Special Issue helped define several molecular mechanisms that characterize GBM, enabling us to focus our efforts on effective and up-to-date topics, with the aim to improve diagnosis, prognosis, and therapy of such a dismal disease.

## References

[1] Stupp R., Mason W.P., van den Bent M.J., Weller M., Fisher B., Taphoorn M.J.B., Belanger K., Brandes A.A., Marosi C., Bogdahn U. (2005). Radiotherapy plus concomitant and adjuvant temozolomide for glioblastoma. N. Engl. J. Med..

[2] Hegi M.E., Diserens A.C., Gorlia T., Hamou M.F., de Tribolet N., Weller M., Kros J.M., Hainfellner J.A., Mason W., Mariani L. (2005). MGMT gene silencing and benefit from temozolomide in glioblastoma. N. Engl. J. Med..

[3] Furnari F.B., Cloughesy T.F., Cavenee W.K., Mischel P.S. (2015). Heterogeneity of epidermal growth factor receptor signalling networks in glioblastoma. Nat. Rev. Cancer.

[4] Meyer M., Reimand J., Lan X., Head R., Zhu X., Kushida M., Bayani J., Pressey J.C., Lionel A.C., Clarke I.D. (2015). Single cell-derived clonal analysis of human glioblastoma links functional and genomic heterogeneity. Proc. Natl. Acad. Sci. USA.

[5] Couturier C.P., Ayyadhury S., Le P.U., Nadaf J., Monlong J., Riva G., Allache R., Baig S., Yan X., Bourgey M. (2020). Single-cell RNA-seq reveals that glioblastoma recapitulates a normal neurodevelopmental hierarchy. Nat. Commun..

[6] Mauldin I.S., Jo J., Wages N.A., Yogendran L.V., Mahmutovic A., Young S.J., Lopes M.B., Slingluff C.L., Erickson L.D., Fadul C.E. (2021). Proliferating CD8+ T Cell Infiltrates Are Associated with Improved Survival in Glioblastoma. Cells.

[7] Vázquez Cervantes G.I., Pineda B., Ramírez Ortega D., Salazar A., González Esquivel D.F., Rembao D., Zavala Vega S., Gómez-Manzo S., Pérez de la Cruz G., Pérez de la Cruz V. (2021). Kynurenine Monooxygenase Expression and Activity in Human Astrocytomas. Cells.

[8] Kraboth Z., Kajtár B., Gálik B., Gyenesei A., Miseta A., Kalman B. (2021). Involvement of the Catecholamine Pathway in Glioblastoma Development. Cells.

[9] Venkatesh H.S., Morishita W., Geraghty A.C., Silverbush D., Gillespie S.M., Arzt M., Tam L.T., Espenel C., Ponnuswami A., Ni L. (2019). Electrical and synaptic integration of glioma into neural circuits. Nature.

[10] Venkataramani V., Tanev D.I., Strahle C., Studier-Fischer A., Fankhauser L., Kessler T., Körber C., Kardorff M., Ratliff M., Xie R. (2019). Glutamatergic synaptic input to glioma cells drives brain tumour progression. Nature.

[11] Fischer I., Nickel A.-C., Qin N., Taban K., Pauck D., Steiger H.-J., Kamp M., Muhammad S., Hänggi D., Fritsche E. (2020). Different Calculation Strategies Are Congruent in Determining Chemotherapy Resistance of Brain Tumors In Vitro. Cells.

[12] Persico M., Abbruzzese C., Matteoni S., Matarrese P., Campana A.M., Villani V., Pace A., Paggi M.G. (2022). Tackling the Behavior of Cancer Cells: Molecular Bases for Repurposing Antipsychotic Drugs in the Treatment of Glioblastoma. Cells.

[13] Hanahan D., Weinberg R.A. (2000). The hallmarks of cancer. Cell.

[14] Hanahan D., Weinberg R.A. (2011). Hallmarks of cancer: The next generation. Cell.

[15] Batara D.C.R., Choi M.-C., Shin H.-U., Kim H., Kim S.-H. (2021). Friend or Foe: Paradoxical Roles of Autophagy in Gliomagenesis. Cells.

[16] Lange F., Hörnschemeyer J., Kirschstein T. (2021). Glutamatergic Mechanisms in Glioblastoma and Tumor-Associated Epilepsy. Cells.

[17] Zhang W., Lin Y. (2021). The Mechanism of Asparagine Endopeptidase in the Progression of Malignant Tumors: A Review. Cells.

[18] Shi Y., Kong Z., Liu P., Hou G., Wu J., Ma W., Cheng X., Wang Y. (2021). Oncogenesis, Microenvironment Modulation and Clinical Potentiality of FAP in Glioblastoma: Lessons Learned from Other Solid Tumors. Cells.

